# Effect of thermal radiation on unsteady magneto-hybrid nanofluid flow in a π -shaped wavy cavity saturated porous medium

**DOI:** 10.3389/fchem.2024.1441077

**Published:** 2024-11-15

**Authors:** A. M. Rashad, Lioua Kolsi, M. A. Mansour, T. Salah, Ahmed Mir, Taher Armaghani, Badr M. Alshammari

**Affiliations:** ^1^ Department of Mathematics, Faculty of Science, Aswan University, Aswan, Egypt; ^2^ Department of Mechanical Engineering, College of Engineering, University of Ha’il, Ha’il City, Saudi Arabia; ^3^ Department of Mathematics, Faculty of Science, Assiut University, Assiut, Egypt; ^4^ Basic and Applied Sciences Department, College of Engineering and Technology, Arab Academy for Science and Technology and Maritime Transport (AASTMT), Aswan, Egypt; ^5^ Department of Chemical and Materials Engineering, College of Engineering, Northern Border University, Arar, Saudi Arabia; ^6^ Department of Engineering, Islamic Azad University, Tehran, Iran; ^7^ Department of Electrical Engineering, College of Engineering, University of Ha’il, Ha’il City, Saudi Arabia

**Keywords:** thermal radiation, wavy-walled, MHD, natural convection, hybrid-nanofluid, porous medium

## Abstract

The present investigation deals with the natural convection (NC) of Al_2_O_3_-Cu-water hybrid nanofluid (HNF) within a “ π ”-shaped cavity under the influence of an externally applied magnetic field (MF). Also we studied the porous media with radiative effect as well as common heat transfer for better fitting to real industrial problems. The inverse U shaped-cavity design includes upper walls that are partially heated and wavy right and left walls designed for cooling purposes, while the remaining walls are maintained as adiabatic. A FORTRAN home code using finite difference method-based approach is adopted to solve the governing equations. A verification is performed by comparing with previous numerical investigations to substantiate the precision of the established numerical model. The findings are expressed in term of stream function, isotherms, and local and averaged Nusselt number. It was found that by increasing amplitude (A), location of the heater (D), thermal radiation parameter (Rd) and wavelength (λ) about 140%, 94%, 775%, and 28% N_uavg_ increases, respectively. In addition, by increasing Dimensionless of heat source/sink length (B), Ha, and heat generation/absorption coefficient (Q) about 20%, 1.1% and 28% N_uavg_ decreases, respectively. Also, N_uavg_ first decreases and then increases by increasing Ra.

## 1 Introduction

The natural convection (NC) mechanism exists in various natural operations such as weather processes, evaporation, and condensation. It is also used in engineering sciences such as cooling systems (Liu et al., 2022), thermal storage (Huang et al., 2022), solar power receivers (Oyewola et al., 2021) and designing buildings (Mikhailenko et al., 2021). Using Nanofluids (NF) and Nanotechology can solve low heat transfer and energy transport in some industrial problems (Zhu et al., 2021; Huang et al., 2020; Ding et al., 2020). A hybrid nanofluid (HNF) is a NF that has at least two kinds of nanoparticles (NPs) suspended in a base fluid leading to more enhanced properties compared to a mono NF (Wang et al., 2022). Some researchers have been published in the field of HNFs (Bantan et al., 2023; Sepehrnia et al., 2022a; Sepehrnia et al., 2023; Sepehrnia et al., 2022b).

Several studies have been conducted on the NC in a porous cavity (PC) with the application of a MF. Jino and Kummar (Jino and Kumar, 2021) worked on the MHD copper-water-NF convective flow in a square PC. The applied MF generates a Lorentz force, which acts on the fluid and opposes the convective motion and heat transfer. Hashemi-Tilehnoee et al. (2020) considered the MHD convection and entropy production in an incinerator filled with Al_2_O_3_-water-NF. The results showed that for fixed Ra value, when the magnitude of the magnetic field is increased, the HT is decreased by 6.28%, while the entropy production is increased by about 31%. Usman et al. (2019) proposed investigating the effects of HT on fluid when a magnetic field is present in a closed square cavity with multiple obstacles. Zhang et al. (2017) considered the coupled effect of thermal radiation and on magneto-natural convective heat transfer in a porous cavity. The findings revealed that with the increase of thermal radiation inside the cavity, the NCHT across its width increased, but with the intensification of the MF magnitude, the HT rate is decreased. Li et al. (2018) studied the NCHT under thermal radiation effect in an enclosure containing iron oxide NPs dispersed in water. Ahmed et al. (2014a) proposed the investigation of NCHT in an inclined PC and conduct heat with a heater in the corner. Alluguvelli et al. (2020) used the FEM to investigate the NCHT in ethylene glycol NF-filled PC. Recently, numerous investigations have been undertaken to explore the MHD coupled convective and radiative heat transfers within porous cavities (PC). Massoudi and Ben Hamida (2020) conducted a simulation using COMSOL software, modeling the behavior of a diamond-water nanofluid within a trapezoidal enclosure featuring elliptical baffles. Ahmed et al. (2014b) provided insights into the impacts of radiation on heat transfer within a porous medium embedded in a cavity, under the effect of an external MF. Sheikholeslami et al. (2018) delved into the simulation of the coupled radiation-convection. The study employed the finite element approach, where the outcomes illuminated that increasing the permeability led to important enhancements in the Nusselt number. Sivaraj and Sheremet (2016) adopted the finite volume numerical approach to study the combined convection-radiation within a porous enclosure. Sivaraj and Sheremet (2017) explored the impact of the MF angle and PM permeability on the HT in a square porous cavity. Employing the finite volume method in their numerical analysis, the results unveiled an inversely correlated relationship between heat transfer and MF strength. Babazadeh et al. (2021) investigated HNF HT in an impermeable cavity that is also affected by an MF. Younis et al. (2022) investigated SWCNTs–water NF HT inside a semi-circular PC in the presence of an MF. The findings revealed that the shorter length of the heated area on the wall enhanced the NCHT. Also, with the growth of R_d_ and Ha, a 4% increase and a 56.5% decrease in the Nu_avg_ obtained, respectively. Rashad et al. (2018) considered the HT of Cu-water NF in a porous medium under the influence of a MF. The confirmed experimental relationships were used to evaluate the properties of the NF. The findings demonstrated that when VF grows, the Nu_avg_ drops. In another study, Sreedevi and Reddy (2022) examined the NCHT in a porous 2D enclosure subjected to the impacts of the thermal radiation source and MF. Amine et al. (2021) investigated NCHT of MgO-Ag-water HNF in a triangular PC under the influence of MF. As the permeability of the PM grew, so did the efficiency of HT. Ghalambaz et al. (2019) investigated NCHT of MWCNTs-MgO-EG HNF in a porous cavity under MF and radiation. It was demonstrated that rising the VF of NF, decreased the HT rate. Dogonchi et al. (2021) considered a closed 2D enclosure with a cylindrical barrier to study the NCHT of NF. Navier-Stokes equations were used to examine the effect of R_d_ and VF on HT. The results showed that the Nu_avg_ increased as the parameters R_d_ and VF grew. Izadi et al. (2019) investigated the unsteady MHD NCHT of a HNF. The impacts of Ra and MF demonstrated on HT. The outcomes demonstrated that as the MF intensity grew, so did the HT. Some other related papers can be seen at Ref. Uma Devi Sathyanarayanan et al. (2021), Mohanty et al. (2021), Pattnaik and Mishra (2020), Mohanty et al. (2019), Pattnaik et al. (2019).

Finally in Table 1, the studies related to the present work are summarized to clarify the differences between the current work and the studies of other researchers. In the present study, NCHT has been carried out inside a π -shaped square wavy PC. As a literature the novelties of current work are: study the heat transfer of HNF in new configuration (inverse U shaped cavity with wavy wall) + unsteady + MHD + porous with radiation. These types of problems such as current work study the future candidate for better cooling process of electronic devises.

**TABLE 1:** Overview of the papers on natural convection.

Authors	Geometry	Nanofluid	MHD	Porous	Radiation
Jino and Kumar (2021)	Square	Cu-water nanofluid	✓	✓	✗
Hashemi-Tilehnoee et al. (2020)	Incinerator shaped	Al_2_O_3_– water nanofluid	✓	✓	✗
Usman et al. (2019)	Square with obstacles	-	✓	✗	✓
Zhang et al. (2017)	Square and Cubic	Magnetic fluid	✓	✗	✓
Li et al. (2018)	Sinusoidal annulus	Fe_3_O_4_– water nanofluid	✓	✗	✓
Ahmed et al. (2014a)	Inclined square	Air	✗	✓	✓
Alluguvelli et al. (2020)	Square	Fe_3_O_4_– E.G., nanofluid	✗	✓	✓
Sivaraj and Sheremet (2016)	Square	Fluid-saturated	✓	✓	✓
Ahmed et al. (2014b)	Square	Fluid-saturated	✓	✓	✓
Massoudi and Ben Hamida (2020)	Trapezoidal	Diamond–water nanofluid	✓	✓	✓
Sheikholeslami et al. (2018)	Complex shaped	Water	✓	✓	✓
Sivaraj and Sheremet (2017)	Inclined square	Fluid-saturated	✓	✓	✓
Babazadeh et al. (2021)	Complex shaped	Fe_3_O_4_-MWCNT-water HNF	✓	✓	✓
Younis et al. (2022)	Semicircular	SWCNTs–water nanofluid	✓	✓	✓
Rashad et al. (2018)	Inclined square	Cu-water nanofluid	✓	✓	✓
Sreedevi and Reddy (2022)	Square	TiO_2_-EG nanofluid	✓	✓	✓
Amine et al. (2021)	Right-angled triangular with a PM quarter-circle shape	Ag-MgO-water HNF	✓	✓	✓
Ghalambaz et al. (2019)	Complex shaped	MgO-MWCNTs-EG hybrid nanofluid	✓	✓	✓
Dogonchi et al. (2021)	Crown wavy	Al_2_O_3_– water nanofluid	✓	✓	✓
Izadi et al. (2019)	Square	Fe_3_O_4_-MWCNT-water HNF	✓	✓	✓
Present work	Inverse U-shaped wavy	Al_2_O_3_-Cu-water HNF	✓	✓	✓

## 2 Mathematical formulation

**FIGURE 1:**
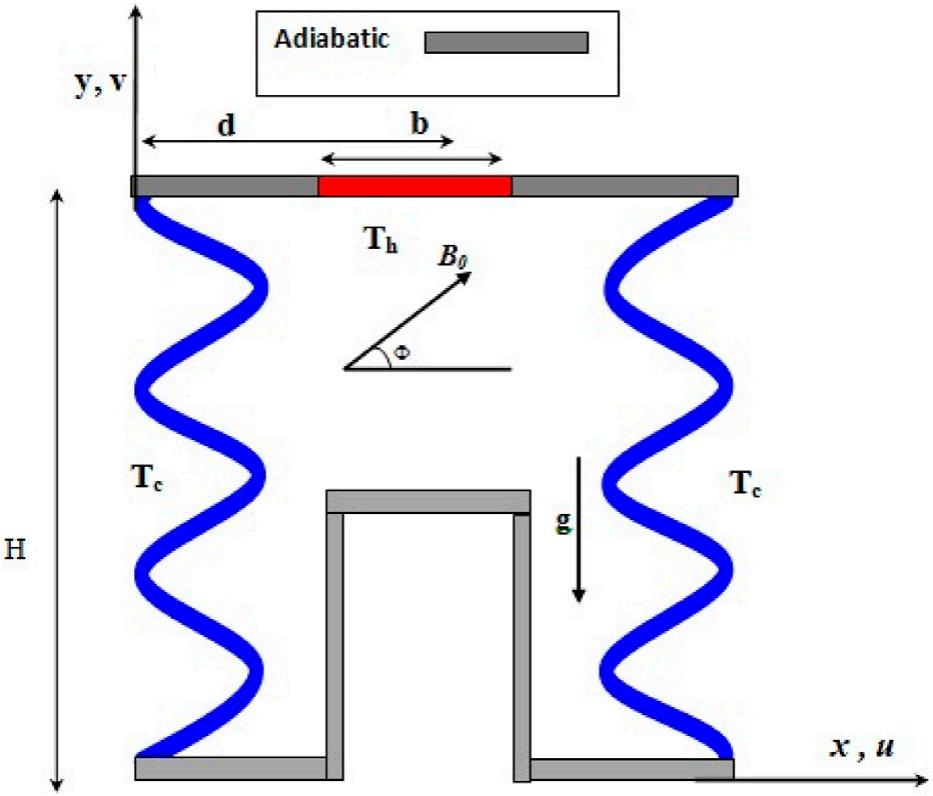
Considered configuration.

The considered configuration is illustrated in Figure 1, featuring an inclined π -shaped cavity ( $\alpha =45^{0}$ ) containing Al_2_O_3_-Cu-water HNF saturated PM. The cavity comprises two wavy walls of wavelength λ, with the right and left walls being cold sinusoidal T_C_. Certain areas in the upper side wall (of length b) are heated with T_h_ (where T_h_ > T_C_), while the remaining walls are considered adiabatic. A MF (B_0_) with an angle Փ in the horizontal orientation acts on the flow. The flow is considered to be Newtonian, laminar, unsteady, No viscous dissipation, No chemical reaction and incompressible. Single phase approach is used for modeling of NHF heat transfer. Nanoparticles and base fluid are in thermal equilibrium. Table 2 lists the properties of both the water and NPs.

**TABLE 2:** Thermophysical properties of water, copper, and Alumina NPs (Rashad et al., 2018; Dogonchi et al., 2021).

Properties	Water	Cu	Al_2_O_3_
ρ [kg.m^-1^]	997.1	8,933	3,970
$C_{p}$ [kJ.kg^-1^.^o^C^-1^]	4.179	0.385	0.765
k [W.m^-1^]	0.613	401	40
β [K^−1^]	21 × 10^−5^	1.67 × 10^−5^	0.85 × 10^−5^
σ [S.m^-1^]	0.05	5.96 × 10^7^	1 × 10^−10^

The governing equations employed in this research are derived from the Boussinesq approximation, which posits that the variation in density within the nanofluid (NF) is essentially negligible, except when considering the influence of buoyancy forces (Rashad et al., 2018).

$$\frac{\partial u}{\partial x}+\frac{\partial v}{\partial y}=0,$$ (1)

$$\frac{1}{\varepsilon^{2}}\left(\frac{\partial u}{\partial t}+u\frac{\partial u}{\partial x}+v\frac{\partial u}{\partial y}\right)=-\frac{1}{\rho_{hnf}}\frac{\partial p}{\partial x}+\frac{\nu_{nf}}{\varepsilon }\left(\frac{\partial^{2}u}{\partial x^{2}}+\frac{\partial^{2}u}{\partial y^{2}}\right)+g\beta_{hnf}\left(T-T_{c}\right)\sin\alpha -\frac{\nu_{hnf}}{K}u+\frac{\sigma_{hnf}B_{0}^{2}}{\rho_{hnf}}\left(v\sin\Phi \cos\Phi -u\sin^{2}\Phi \right),$$ (2)

$$\frac{1}{\varepsilon^{2}}\left(\frac{\partial v}{\partial t}+u\frac{\partial v}{\partial x}+v\frac{\partial v}{\partial y}\right)=-\frac{1}{\rho_{nf}}\frac{\partial p}{\partial y}+v_{hnf}\left(\frac{\partial^{2}v}{\partial x^{2}}+\frac{\partial^{2}v}{\partial y^{2}}\right)+g\beta_{hnf}\left(T-T_{c}\right)\cos\alpha -\frac{\nu_{hnf}}{K}v+\frac{\sigma_{hnf}B_{0}^{2}}{\rho_{hnf}}\left(u\sin\Phi \cos\Phi -v\cos^{2}\Phi \right),$$ (3)

$$\frac{1}{\varepsilon }\left(\frac{\partial T}{\partial t}+u\frac{\partial T}{\partial x}+v\frac{\partial T}{\partial y}\right)=\left[\alpha_{eff.nf}\right.+\left.\frac{16\sigma^{*}T_{c}^{3}}{3k^{*}{\left(\rho c_{p}\right)}_{hnf}}\right].\nabla^{2}T+\frac{Q_{0}}{\varepsilon {\left(\rho c_{p}\right)}_{hnf}},$$ (4)

The imposed initial and boundary conditions are (Alsabery et al., 2021):

$$\begin{array}{c} t≺0:u=v=T=0,0\leq x\leq H,0\leq y\leq H,\\ \;t\geq 0:u=v=0,0\leq y\leq H,0\leq x\leq H\;at\;all\;walls,\\ T=T_{H},D-0.5B\leq \frac{y}{H}\leq D+0.5B,\\ \frac{\partial T}{\partial x}=0,else\;at\;wall\;x=1\\ T=T_{c},x=H-AH\left[1-\cos\left(\frac{2\pi \lambda y}{H}\right)\right],0\leq y\leq H\\ T=T_{c}x=AH\left[1-\cos\left(\frac{2\pi \lambda y}{H}\right)\right],0\leq y\leq H \end{array}$$ (5)

Multiple formulations for the thermophysical properties of NFs have been put forth in existing literature. However, in this investigation, the employed relationships solely rely on the VF and have been validated and employed in prior research endeavors by Khanafer et al. (2003) and Brinkman (1952).

The thermal diffusivities of an HNF ( $\alpha_{eff,nf}$ ) and a PM ( $\alpha_{eff,f}$ ) can be illustrated through the use of two following Equations 6 and 7:

$\alpha_{eff,Hnf}$ and $\alpha_{eff,f}$ illustrate the efficient thermal diffusion of HNF and PM and, likewise, the efficient thermal diffusion of the base fluid and PM. These values are equal to:

$$\alpha_{eff,nf}=\frac{k_{eff,hnf}}{{\left(\rho c_{p}\right)}_{hnf}}$$ (6)

$$\alpha_{eff,f}=\frac{k_{eff,f}}{{\left(\rho c_{p}\right)}_{f}}$$ (7)

The effective thermal conductivity (k_eff, hnf_) of an HNF and a PM can be determined by using a specific equation (Rashad et al., 2018).

$$k_{eff,nf}=\varepsilon k_{hnf}+\left(1-\varepsilon \right)k_{s}$$ (8)

The k_eff,hnf_ of a PM can be determined based on the solid thermal conductivity (k_s_) and the porosity (ε) of the PM. This can be expressed mathematically using the following equation.

$$k_{eff,f}=\varepsilon k_{f}+\left(1-\varepsilon \right)k_{s}$$ (9)

It is noteworthy to mention that the thermal conductivities of the HNF and the PM have been treated as highly similar in depicting the outcomes, according to the thermal equilibrium assumption presented in Equations 8, 9.

### 2.1 Thermophysical properties of NF and HNF

Although previous research has made efforts to ascertain the thermophysical characteristics of Nanofluids (NFs), the conventional models employed in these investigations have shown limited accuracy when applied to NFs. Nevertheless, empirical data can aid in selecting an appropriate model for a particular property. The effective characteristics of two specific Nanofluids, specifically, Al_2_O_3_-water and Al_2_O_3_-Cu-water Hybrid Nanofluids (HNFs), can be articulated as follows:

$$\rho_{nf}=\left(1-\phi \right)\rho_{bf}+\phi \rho_{p}$$ (10)

Equation 10 is used to determine the density of NFs. Consequently, the density of HNF is defined as follows in Equation 11:

$$\rho_{nf}=\phi_{Al_{2}O_{3}}\rho_{Al_{2}O_{3}}+\phi_{Cu}\rho_{Cu}+\left(1-\phi \right)\rho_{bf},$$ (11)

where $\phi =\phi_{Al_{2}O_{3}}+\phi_{Cu}$ ,

The heat capacity of the NF is expressed as Khanafer et al. (2003):

$${\left(\rho C_{p}\right)}_{nf}=\phi {\left(\rho C_{p}\right)}_{p}+\left(1-\phi \right){\left(\rho C_{p}\right)}_{bf}$$ (12)

Referring to Equation 12, the heat capacity of HNF can be calculated in the subsequent manner Presented at Equation 13:

$${\left(\rho C_{p}\right)}_{nf}=\phi_{Al_{2}O_{3}}{\left(\rho C_{p}\right)}_{Al_{2}O_{3}}+\phi_{Cu}{\left(\rho C_{p}\right)}_{Cu}+\left(1-\phi \right){\left(\rho C_{p}\right)}_{bf}$$ (13)

Other thermal properties of nanofluid and hybrid nanofluid can be seen at Equations 14–23. The thermal expansion coefficient of the NF can be obtained through the equation:

$${\left(\rho \beta \right)}_{nf}=\phi {\left(\rho \beta \right)}_{p}+\left(1-\phi \right){\left(\rho \beta \right)}_{bf}$$ (14)

Therefore, for HNF, thermal expansion is describable in the subsequent manner:

$${\left(\rho \beta \right)}_{nf}=\phi_{Al_{2}O_{3}}{\left(\rho \beta \right)}_{Al_{2}O_{3}}+\phi_{Cu}{\left(\rho \beta \right)}_{Cu}+\left(1-\phi \right){\left(\rho \beta \right)}_{bf}$$ (15)

Thermal diffusivity, $\alpha_{nf}$ of the NF is expressed as Oztop and Abu-Nada (2008):

$$\alpha_{nf}=\frac{k_{nf}}{{\left(\rho c_{p}\right)}_{nf}}$$ (16)

The thermal conductivity of the NF is (Maxwell, 1873):

$$\frac{k_{nf}}{k_{bf}}=\frac{\left(k_{p}+2k_{bf}\right)-2\phi \left(k_{bf}-k_{p}\right)}{\left(k_{p}+2k_{bf}\right)+\phi \left(k_{bf}-k_{p}\right)}$$ (17)

Therefore, the thermal diffusivity, $\alpha_{nf}$ of the HNF is:

$$\alpha_{nf}=\frac{k_{hnf}}{{\left(\rho C_{p}\right)}_{hnf}},$$ (18)

The thermal conductivity of the HNF is expressed as:

$$\frac{k_{nf}}{k_{bf}}=\left(\frac{\left(\phi_{Al_{2}O_{3}}k_{Al_{2}O_{3}}+\phi_{Cu}k_{Cu}\right)}{\phi }+2k_{bf}+2\left(\phi_{Al_{2}O_{3}}k_{Al_{2}O_{3}}+\phi_{Cu}k_{Cu}\right)-2\phi k_{bf}\right)\times {\left(\frac{\left(\phi_{Al_{2}O_{3}}k_{Al_{2}O_{3}}+\phi_{Cu}k_{Cu}\right)}{\phi }+2k_{bf}-\left(\phi_{Al_{2}O_{3}}k_{Al_{2}O_{3}}+\phi_{Cu}k_{Cu}\right)+\phi k_{bf}\right)}^{-1}$$ (19)

The dynamic viscosity of the NF and HNF are expressed as Mohanty et al. (2019):

$$\mu_{nf}=\frac{\mu_{bf}}{{\left(1-\phi \right)}^{2.5}}$$ (20)

$$\mu_{nf}=\frac{\mu_{bf}}{{\left(1-\left(\phi_{Al_{2}O_{3}}+\phi_{Cu}\right)\right)}^{2.5}}$$ (21)

the electrical conductivity of the NF and HNF are expressed as Mohanty et al. (2019):

$$\frac{\sigma_{nf}}{\sigma_{bf}}=1+\frac{3\left(\frac{\sigma_{p}}{\sigma_{bf}}-1\right)\phi }{\left(\frac{\sigma_{p}}{\sigma_{bf}}+2\right)-\left(\frac{\sigma_{p}}{\sigma_{bf}}-1\right)\phi }$$ (22)

$$\frac{\sigma_{nf}}{\sigma_{bf}}=1+\frac{3\left(\frac{\left(\phi_{Al_{2}O_{3}}\sigma_{Al_{2}O_{3}}+\phi_{Cu}\sigma_{Cu}\right)}{\sigma_{bf}}-\left(\phi_{Al_{2}O_{3}}+\phi_{Cu}\right)\right)}{\left(\frac{\left(\phi_{Al_{2}O_{3}}\sigma_{Al_{2}O_{3}}+\phi_{Cu}\sigma_{Cu}\right)}{\phi \sigma_{bf}}+2\right)-\left(\frac{\left(\phi_{Al_{2}O_{3}}\sigma_{Al_{2}O_{3}}+\phi_{Cu}\sigma_{Cu}\right)}{\sigma_{bf}}-\left(\phi_{Al_{2}O_{3}}+\phi_{Cu}\right)\right)}$$ (23)

These thermal properties equations of HNF and NF also used in some related papers (Pattnaik et al., 2019; Alsabery et al., 2021; Khanafer et al., 2003).

Introducing the subsequent dimensionless variables:

$$X=\frac{x}{H},Y=\frac{y}{H},U=\frac{uH}{\alpha_{f}},V=\frac{vH}{\alpha_{f}},P=\frac{pH^{2}}{\rho_{nf}\alpha_{f}^{2}},\theta =\frac{T-T_{c}}{T_{h}-T_{c}},\tau =\frac{\alpha_{f}}{H^{2}}t$$ (24)

into Equations 1–5 gives rise to the dimensionless set of equations:

$$\frac{\partial U}{\partial X}+\frac{\partial V}{\partial Y}=0$$ (25)

$$\frac{1}{\varepsilon^{2}}\left(\frac{\partial U}{\partial \tau }+U\frac{\partial U}{\partial X}+V\frac{\partial U}{\partial Y}\right)=-\frac{\partial P}{\partial X}+\frac{v_{nf}}{\varepsilon v_{f}}\mathrm{Pr}\left(\frac{\partial^{2}U}{\partial X^{2}}+\frac{\partial^{2}U}{\partial Y^{2}}\right)+\text{Ra}\frac{\beta_{hnf}}{\beta_{f}}\mathrm{Pr}\theta \sin\alpha -\frac{v_{hnf}}{v_{f}}\frac{\mathrm{Pr}}{Da}+Ha^{2}.\mathrm{Pr}.\frac{\sigma_{hnf}}{\sigma_{f}}\left(V\sin\Phi \cos\Phi -U\sin^{2}\Phi \right)$$ (26)

$$\frac{1}{\varepsilon^{2}}\left(\frac{\partial V}{\partial \tau }+U\frac{\partial V}{\partial X}+V\frac{\partial V}{\partial Y}\right)=-\frac{\partial P}{\partial Y}+\frac{v_{nf}}{\varepsilon v_{f}}\mathrm{Pr}\left(\frac{\partial^{2}V}{\partial X^{2}}+\frac{\partial^{2}V}{\partial Y^{2}}\right)+\text{Ra}\frac{\beta_{hnf}}{\beta_{f}}\mathrm{Pr}\theta \cos\alpha -\frac{v_{hnf}}{v_{f}}\frac{\mathrm{Pr}}{Da}+Ha^{2}\mathrm{Pr}\frac{\sigma_{hnf}}{\sigma_{f}}\left(U\sin\Phi \cos\Phi -V\cos^{2}\Phi \right)$$ (27)

$$\frac{1}{\varepsilon }\left(\frac{\partial \theta }{\partial \tau }+U\frac{\partial \theta }{\partial X}+V\frac{\partial \theta }{\partial Y}\right)=\frac{\alpha_{eff.nf}}{\alpha_{eff.f}}\left(1+R_{d}\right)\left(\frac{\partial^{2}\theta }{\partial X^{2}}+\frac{\partial^{2}\theta }{\partial Y^{2}}\right)+\frac{{\left(\rho c_{p}\right)}_{f}}{{\left(\rho c_{p}\right)}_{hnf}}.Q$$ (28)

Where

$$\mathrm{Pr}=\frac{\nu_{f}}{\alpha_{f}},Ra=\frac{g\beta_{f}\left(T_{H}-T_{c}\right)H^{3}}{\alpha_{f}\nu_{f}},Ha=B_{0}H\sqrt{\frac{\sigma_{f}}{\mu_{f}}},R_{d}=\frac{16\sigma^{*}T_{c}^{3}}{3k_{nf}k^{*}},Q=\frac{Q_{0}H^{2}}{{\left(\rho c_{p}\right)}_{f}\alpha_{f}}$$

The dimensionless boundary conditions become:

$$\begin{array}{c} \tau ≺0:U=V=\theta =0,0\leq X\leq 1,0\leq Y\leq 1,\\ \tau \geq 0:U=V=0,0\leq Y\leq 1,0\leq X\leq 1\;at\;all\;walls\;\\ \theta =1,D-0.5B\leq Y\leq D+0.5B,\\ \frac{\partial \theta }{\partial X}=0,otherwise\;at\;wall\;X=1\\ \theta =0,X=1-A\left[1-\cos\left(2\pi \lambda Y\right)\right],0\leq Y\leq 1\;\\ \theta =0,X=A\left[1-\cos\left(2\pi \lambda Y\right)\right],0\leq Y\leq 1 \end{array}$$ (29)

The local Nu is specified as Equation 30:

$$Nu_{s}=-\frac{k_{nf}}{k_{f}}\left(1+R_{d}\right)\left(\frac{\partial \theta }{\partial Y}\right)$$ (30)

And the average Nu is specified as Equation 31:

$$Nu_{m}=\frac{1}{B}\int_{D-0.5*B}^{D+0.5*B}Nu_{s}dX$$ (31)

## 3 Numerical procedure

In this particular investigation, the transient dimensionless governing equations (Equations 25–28) are addressed using the iterative finite difference method, while adhering to the specific boundary conditions described in Equation 29. To consider the directional influence of perturbations, a second-order upwind finite differencing scheme is applied to approximate convective terms.

The finite difference approximation for the heat equation can be represented in Equation 32:

$$\theta_{i,j}^{n+1}=\theta_{i,j}^{n}+\frac{∆\tau }{\sigma }\left\{\left(\frac{\alpha_{nf}}{\alpha_{f}}\right)\left[\frac{\theta_{i+1,j}^{n}-2\theta_{i,j}^{n}+\theta_{i-1,j}^{n}}{{\left(∆X\right)}^{2}}+\frac{\theta_{i,j+1}^{n}-2\theta_{i,j}^{n}+\theta_{i,j-1}^{n}}{{\left(∆Y\right)}^{2}}\right]+Q\frac{{\left({\rho c}_{p}\right)}_{f}}{{\left({\rho c}_{p}\right)}_{nf}}\theta_{i,j}^{n}-U_{i,j}^{n}\frac{\theta_{i+1,j}^{n}-\theta_{i-1,j}^{n}}{2∆X}-V_{i,j}^{n}\frac{\theta_{i,j+1}^{n}-\theta_{i,j-1}^{n}}{2∆Y}\right\}$$ (32)

The cell locations in question are denoted by i and j. Equations 26–28 are subject to a similar approximation method. The next conjunction criteria were employed for parameters that were based on unknowns shown in Equation 33:

$$\sum_{i,j}\left|\chi_{i,j}^{\text{new}}-\chi_{i,j}^{\text{old}}\right|\leq 10^{-6}.$$ (33)

The non-uniform grid contains of 61 × 61 grid nodes in the *X*-and *Y*-directions, respectively. The obtained data are separated of the number of the grids. The grid independency data are displayed in Table 3.

**TABLE 3:** Grid-independency study.

Grid-size	41×41	51×51	61×61	71×71
${Nu}_{m}$	1.469,836	1.458,858	1.437,232	1.43654

**FIGURE 2:**
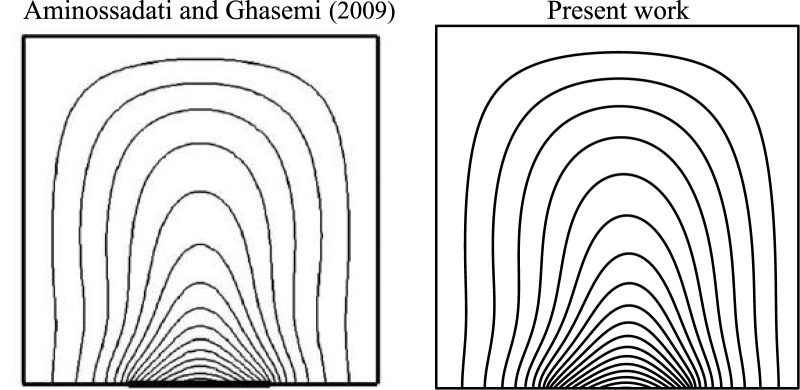
Comparison of the temperature field with the results of Aminossadati and Ghasemi (2009) at *B* = 0.4, $Ra=10^{5},\phi =0.1,D=0.5$ .

These parameters were determined to be adequate for achieving a steady state within a computationally feasible time frame. A FORTRAN home code using finite difference method-based approach is adopted to solve the governing equations. To verify the precision of the current approach, the acquired outcomes were compared with those obtained by Aminossadati and Ghasemi (2009) in particular cases ( B=0.4, ; =10% ). Nu_av_ at the heat source was used as the metric for comparison, and the findings were displayed in Figure 2 and Table 4. A high degree of agreement was observed between the results obtained by the two methods.

**TABLE 4:** Comparison the values of Nu_av_ with the findings of Aminossadati and Ghasemi (Aminossadati and Ghasemi, 2009) fo B=0.4 , ϕ=10%,D = 0.5.

Ra	Aminossadati and Ghasemi (2009)	Present results	Deviation %
10^4^	5.474	5.475	0.018
10^5^	7.121	7.204	1.2
10^6^	13.864	14.014	1.1

## 4 Results and discussion

In this research endeavor, a numerical modeling approach is used to scrutinize the influence of fluctuations in amplitude (A) and wavelength (λ) of sinusoidal wall oscillations, as well as the parameters Ra, Ha, length of the heat source (B), location of the heater (D), heat generation/absorption coefficient (Q), and thermal radiation parameter (Rd) on the characteristics of stream function, isotherms, and the local-average Nusselt number (Nu).

**FIGURE 3:**
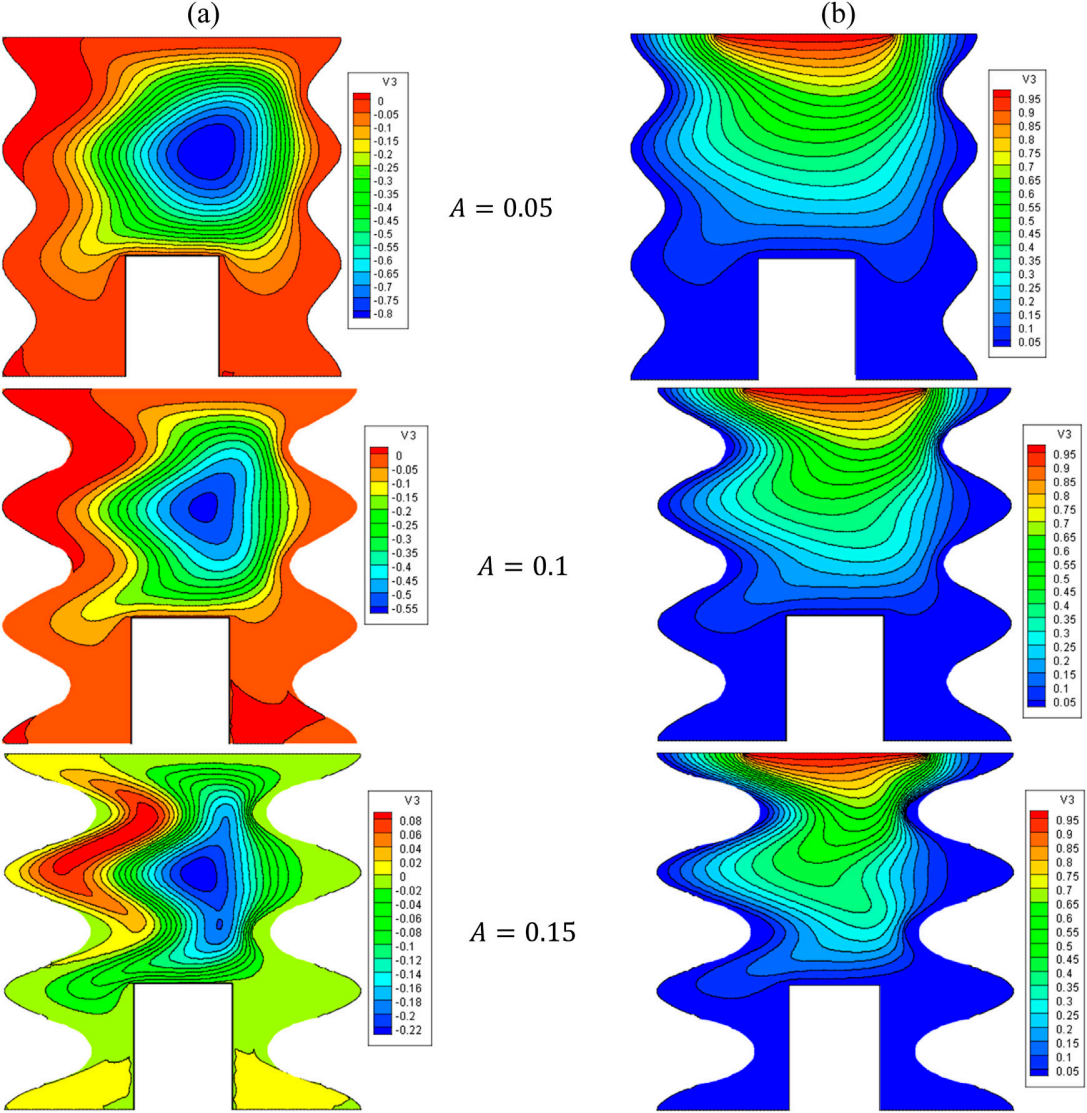
**(A)** Stream function and **(B)** isotherms for HNFs at *Ha* = 10, *φ* = 0.05, *Q* = 1, *D* = 0.5, *R_d_* = 1, *λ* = 3, *α* = 45°, *Ra* = 10^5^, *Φ* = 60°, *φ_Cu_* = *φ_Al_2_O_3__* = *φ*/2.

Figure 3 presents the effects varying the sinusoidal amplitude of the left and right boundaries (A) on the flow structure (stream function) and temperature field (isotherms) for $\text{Ha}=10,\varphi =0.05,Q=1,D=0.5,R_{d}=1,\lambda =3,\alpha =45{}^{\circ},\text{Ra}=10^{5},\Phi =60{}^{\circ},\varphi_{\text{Cu}}=\varphi_{\text{Al}2O3}=\varphi /2$ .

The temperature of Fluid near the hot wall increases so the gravity decreases and the fluid move to top wall. After facing the cold wall the fluid temperature reduces as well as increasing the gravity. So the fluid moves to bottom of cavity and the streamlines appears as a clock wise rotation. Generally this manner can be seen in all of streamlines figures.

The isotherm lines sown very high temperature gradient need the edges of top wall. So the Nu number experiences more values in these areas. As a same this manner can be seen in all of figures about local Nu Number.

**FIGURE 4:**
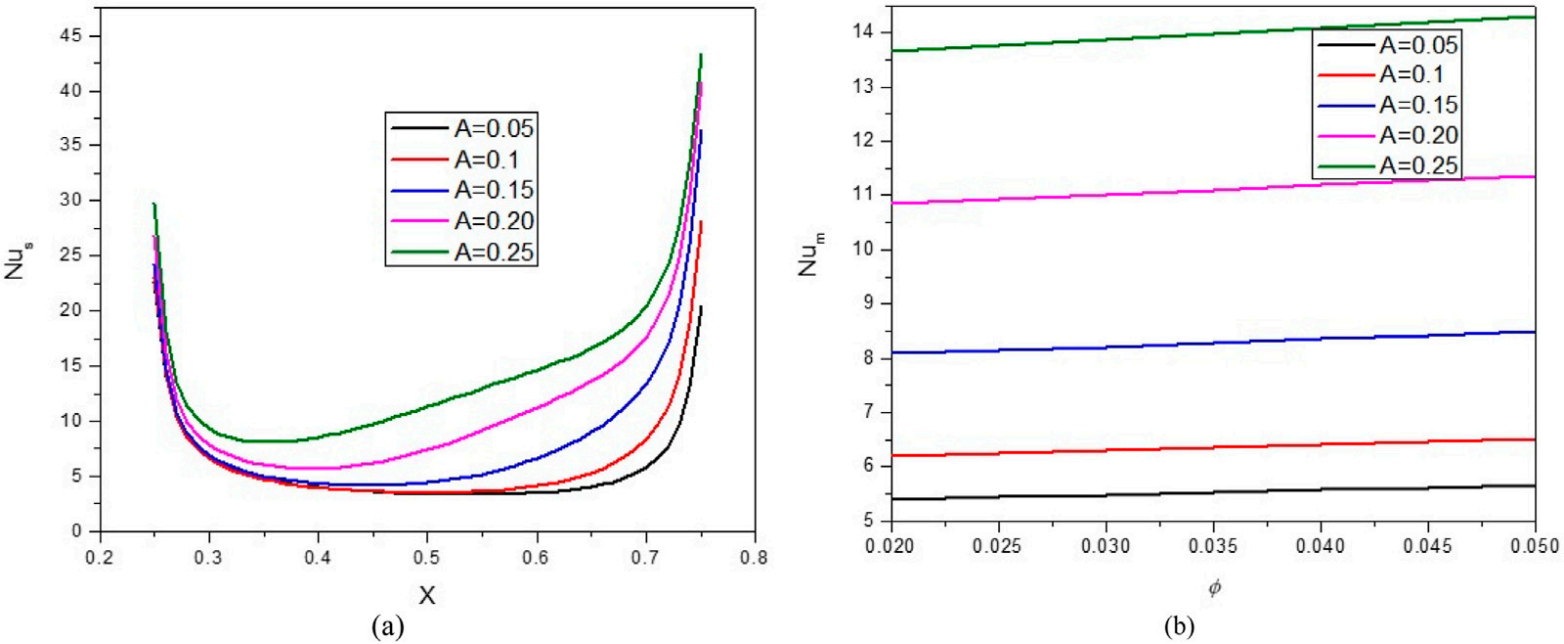
**(A)** Nu and **(B)** Nu_avg_ at *Ha* = 10, *φ* = 0.05, *Q* = 1, *B* = 0.5, *R*_*d*_ = 1, *λ* = 3, *α* = 45^0^, *Ra* = 10^5^, Φ = 60^0^, *φ*_*Cu*_ = *φ*_*Al*_2___*O*_3__ = *φ*/2.

Figure 4 displays the variations of the Nu_local_ versus the A and the Nu_avg_ versus the VF. As can be seen, the stream function and isotherms have become compressed by increasing A. The stream function and isotherms in the cavity slope from top to bottom. These variations give rise to an elevation in the temperature gradient proximate to the active surface, thereby causing a corresponding enhancement in Nu_avg_. In this regard, by increasing A from 0.05 to 0.15, about a 140% increase in the Nu_avg_ is observed. Also, by increasing the VF, HT is improved owing to the rise in the thermal conductivity and the increase in the average temperature in the cavity.

**FIGURE 5:**
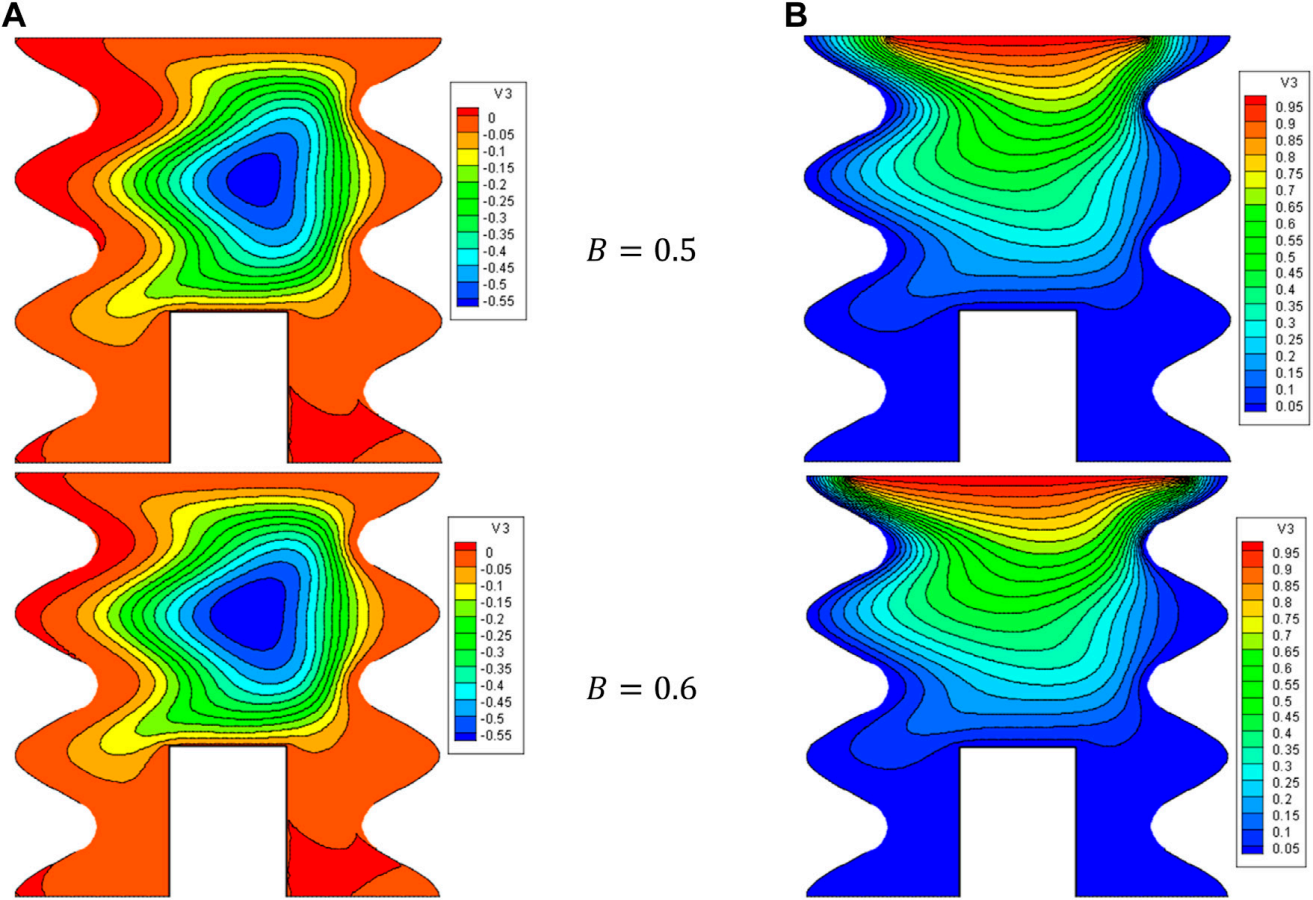
**(A)** Stream function and **(B)** isotherms for HNFs at *Ha* = 10, *φ* = 0.05, *Q* = 1, *D* = 0.5, *R*_*d*_ = 1, *λ* = 3, *α* = 45^0^, *Ra* = 10^5^, Φ = 60^0^, *φ*_*Cu*_ = *φ*_*Al*_2___*O*_3__ = *φ*/2..

**FIGURE 6:**
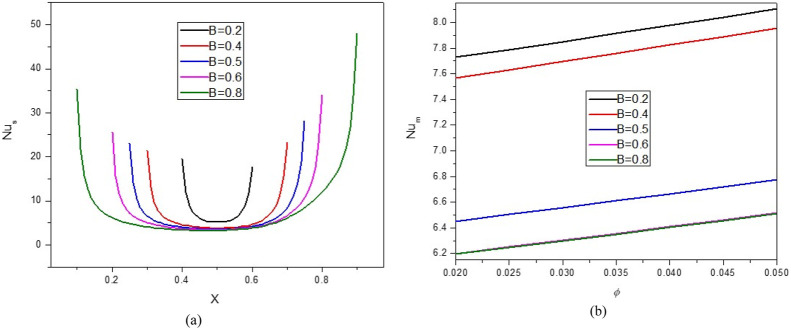
**(A)** Nu and **(B)** Nu_avg_ at *Ha* = 10, *ϕ* = 0.05, *Q* = 1, *B* = 0.5, *R*_*d*_ = 1, *λ* = 3, *α* = 45^0^, *Ra* = 10^5^, Φ = 60^0^, *ϕ*_*Cu*_ = *ϕ*_*Al*_2___*O*_3__ = *ϕ*/2.

Figures 5, 6 illustrate the impact of the B on the stream function, isotherms, and Nu_local_ - Nu_avg_ in specific conditions as follows:

$\text{Ha}=10,\varphi =0.05,Q=1,D=0.5,R_{d}=1,\lambda =3,\alpha =45{}^{\circ},\text{Ra}=10^{5},\Phi =60{}^{\circ},\varphi_{\text{Cu}}=\varphi_{\text{Al}2O3}=\varphi /2$ . The results indicate that reducing B is an effective way to improve NCHT. In other words, reducing B reduces waste, increases the strength of vortices, and increases NCHT in the cavity. Increasing B makes the core of eddy larger. Results show that by increasing B, the Nu_local_ - Nu_avg_ decreases so that when B changes from 0.2 to 0.8, about a 20% decrease in Nu_avg_ is observed. At a constant B, increasing the VF from 2% to 5% increases the Nu_avg_ by about 4.6%.

**FIGURE 7:**
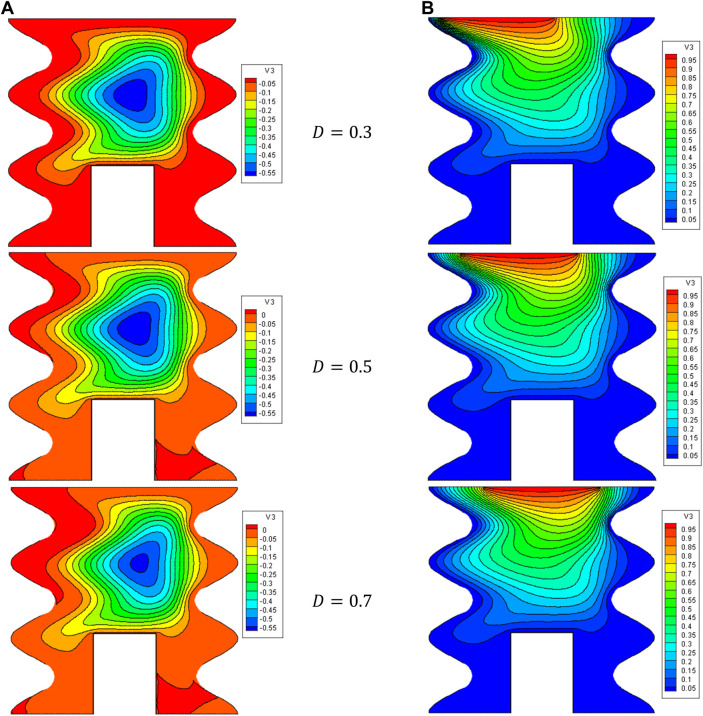
**(A)** Stream function and **(B)** isotherms for HNFs at *Ha* = 10, *φ* = 0.05, *Q* = 1, *B* = 0.5, *R*_*d*_ = 1, *λ* = 3, *α* = 45^0^, *Ra* = 10^5^, Φ = 60^0^, *φ*_*Cu*_ = *φ*_*Al*_2___*O*_3__ = *φ*/2.

**FIGURE 8:**
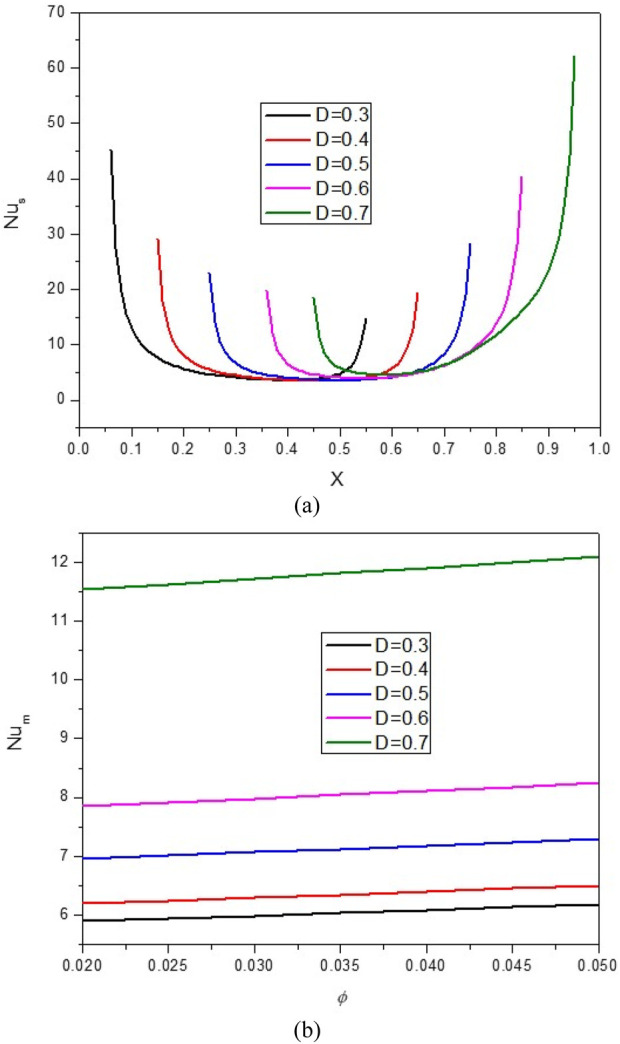
**(A)** Nu and **(B)** Nu_avg_ at *Ha* = 10, *φ* = 0.05, *Q* = 1, *B* = 0.5, *R*_*d*_ = 1, *λ* = 3, *α* = 45^0^, *Ra* = 10^5^, Φ = 60^0^, *φ*_*Cu*_ = *φ*_*Al*_2___*O*_3__ = *φ*/2.

Figures 7, 8 show the impact of the D on the stream function, isotherms, and Nu_local_ - Nu_avg_ in specific conditions as follows:

$$\text{Ha}=10,\varphi =0.05,Q=1,B=0.5,R_{d}=1,\lambda =3,\alpha =45{}^{\circ},\text{Ra}=10^{5},\Phi =60{}^{\circ},\varphi_{\text{Cu}}=\varphi_{\text{Al}2O3}=\varphi /2.$$

The results show that increasing D from the isotherms with a higher temperature towards the center of the cavity can cause NCHT and more flow circulation in the cavity. In fact, by increasing D, the buoyancy force is strengthened, and the HNF can easily overcome the viscous force. In addition, by increasing D, the strength of vortices and convection areas increases, which increases the mean temperature of the HNF in the cavity and increases HT. Following this phenomenon, the Nu_local_ - Nu_avg_ in the cavity improves by increasing D so that when D changes from 0.3 to 0.7, about a 94% increase in Nu_avg_ is observed. Also, at a constant D, increasing the VF from 2% to 5% increases the Nu_avg_ by about 3.7%.

**FIGURE 9:**
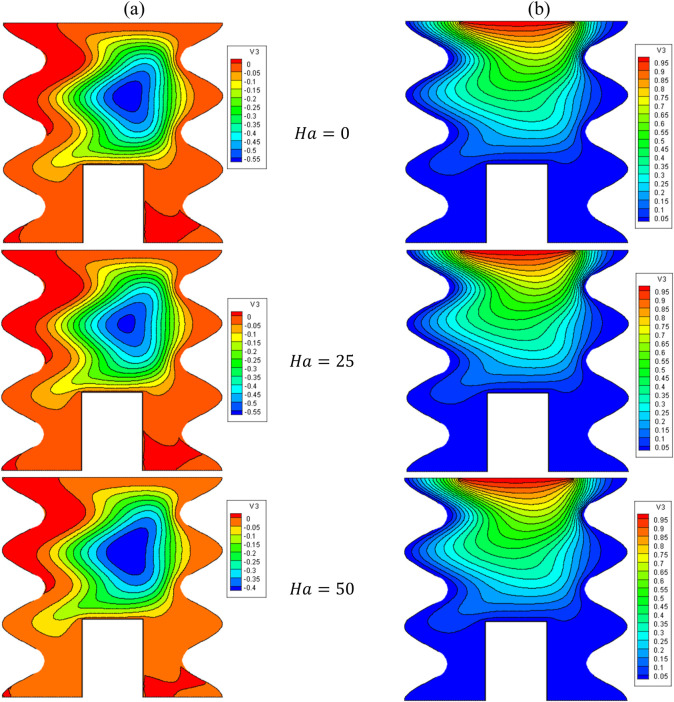
**(A)** Stream function and **(B)** isotherms for HNFs at *φ* = 0.05, *Q* = 1, *B* = 0.5, *D* = 0.5, *R*_*d*_ = 1, *λ* = 3, *α* = 45^0^, *Ra* = 10^5^, Φ = 60^0^, *φ*_*Cu*_ = *φ*_*Al*_2___*O*_3__ = *φ*/2.

**FIGURE 10:**
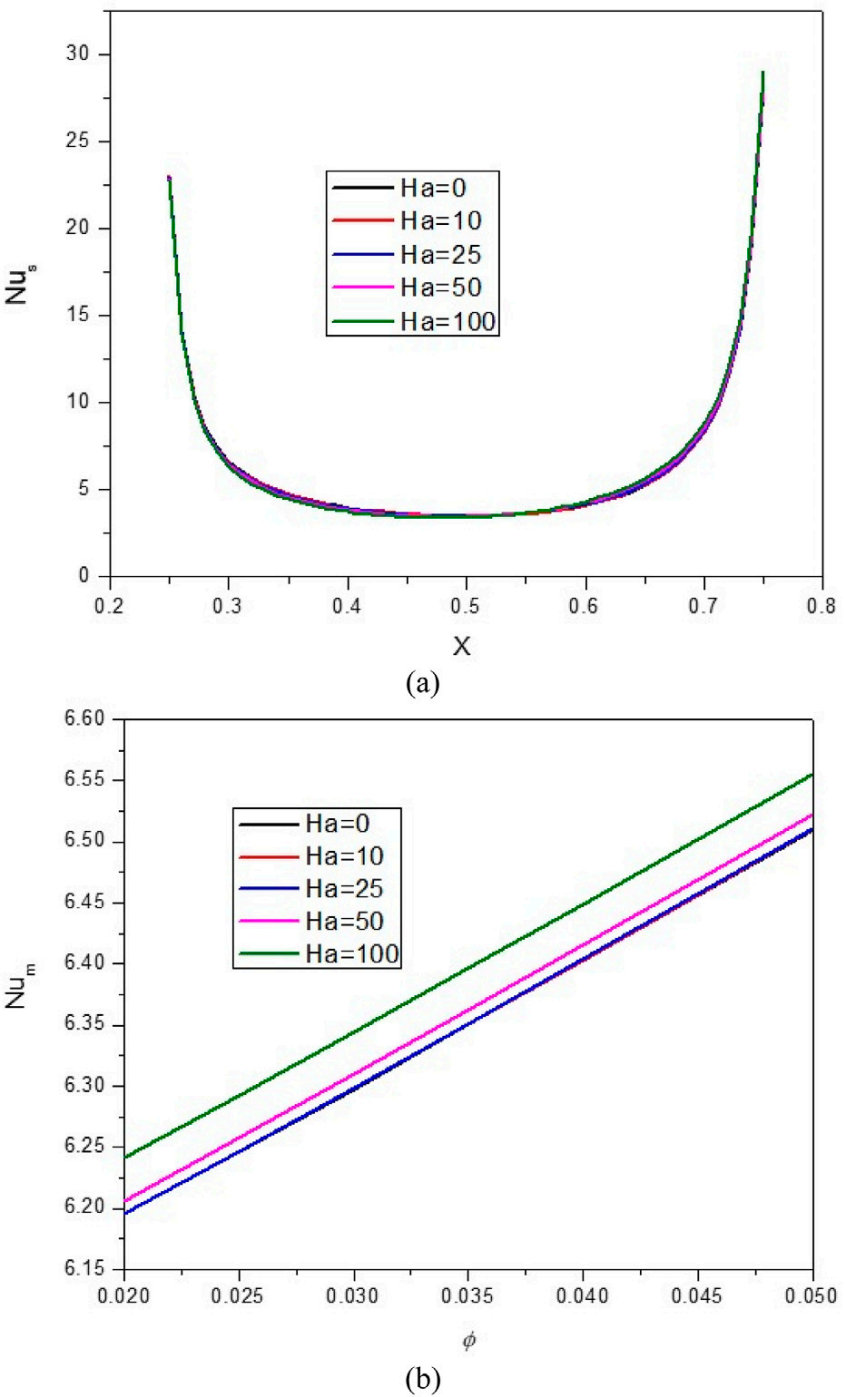
**(A)** Nu and **(B)** Nu_avg_ at *Ha* = 10, *φ* = 0.05, *Q* = 1, *B* = 0.5, *R*_*d*_ = 1, *λ* = 3, *α* = 45^0^, *Ra* = 10^5^, Φ = 60^0^, *φ*_*Cu*_ = *φ*_*Al*_2___*O*_3__ = *φ*/2.

Figures 9, 10 present the impact of Ha on the stream function, isotherms, and Nu_local_ - Nu_avg_ in specific conditions as follows:

$$\varphi =0.05,Q=1,B=D=0.5,R_{d}=1,\lambda =3,\alpha =45{}^{\circ},\text{Ra}=10^{5},\Phi =60{}^{\circ},\varphi_{\text{Cu}}=\varphi_{\text{Al}2O3}=\varphi /2.$$

The magnetic field (MF) induces alterations in the flow patterns by exerting the Lorentz force. When the Lorentz force aligns with the buoyancy force, it promotes an augmentation of Nusselt number and leads to an increase in the average temperature within the enclosure. In contrast, when the Lorentz force counteracts the buoyancy force, it results in a reduction of NCHT. However, it is noteworthy to highlight that in the current investigation, as evident from the stream function and isotherm contours, the application of the MF yields minimal impact on both the flow and temperature patterns. The Nu_local_ - Nu_avg_ changes with increasing Ha show a slight decrease in HT; as the Ha increases from 0 to 100, there is a 1.1% decrease in the Nu_avg_. At a constant Ha, by increasing the VF from 2% to 5%, an average increase of 4.9% in Nu_avg_ is observed.

**FIGURE 11:**
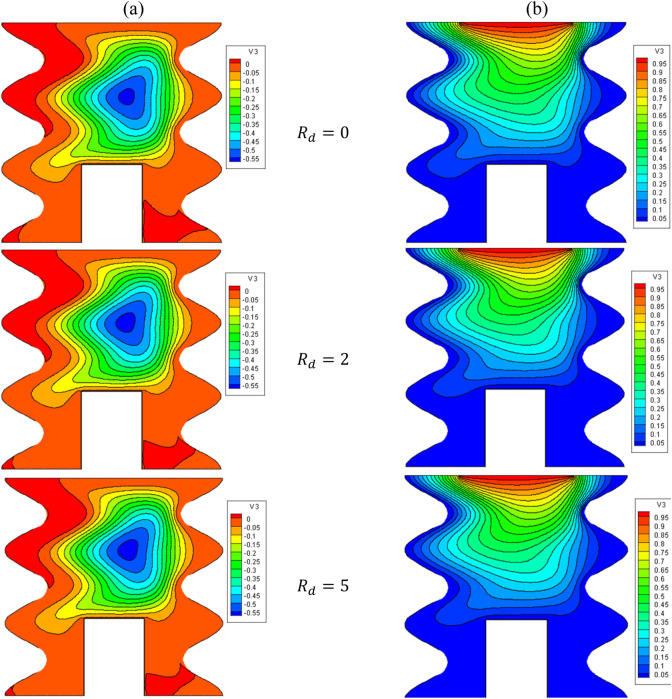
**(A)** Stream function and **(B)** isotherms for HNFs at *φ* = 0.05, *Q* = 1, *B* = 0.5, *D* = 0.5, *Ha* = 10, *λ* = 3, *α* = 45^0^, *Ra* = 10^5^, Φ = 60^0^, *φ*_*Cu*_ = *φ*_*Al*_2___*O*_3__ = *φ*/2.

**FIGURE 12:**
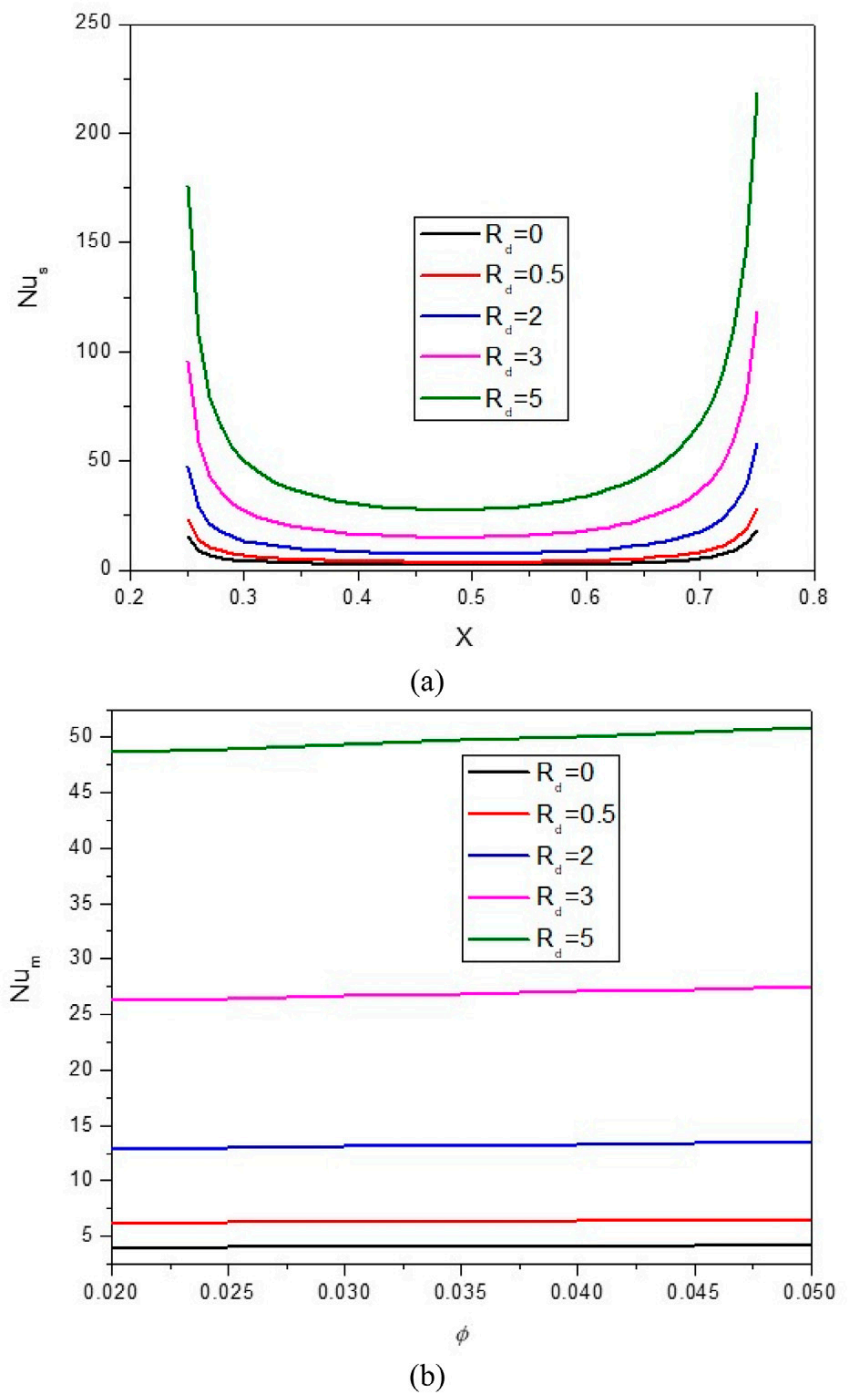
**(A)** Nu and **(B)** Nu_avg_ at *Ha* = 10, *φ* = 0.05, *Q* = 1, *B* = 0.5, *R*_*d*_ = 1, *λ* = 3, *α* = 45^0^, *Ra* = 10^5^, Φ = 60^0^, *φ*_*Cu*_ = *φ*_*Al*_2___*O*_3__ = *φ*/2.

Figures 11, 12 illustrate the impact of the R_d_ on the stream function, isotherms, and Nu_local_ - Nu_avg_ in specific conditions as follows:

$\varphi =0.05,Q=1,B=D=0.5,\text{Ha}=10,\lambda =3,\alpha =45{}^{\circ},\text{Ra}=10^{5},\Phi =60{}^{\circ},\varphi_{\text{Cu}}=\varphi_{\text{Al}2O3}=\varphi /2$ . Increasing R_d_ increases the core size of hot and cold HNF vortices. Thus, it increases mixing and improves HT. As can be seen, by increasing Rd, the Nu_local_ increases. Furthermore, under constant values of the thermal radiation parameter (R_d_), the average Nusselt number (Nu_avg_) experiences a rise as the volume fraction (VF) increases. Therefore, increasing R_d_ positively affects NCHT inside the cavity; so by increasing R_d_ from 0 to 5, the Nu_avg_ becomes about 8.75 times larger. Increasing the VF does not have much influence on strengthening the R_d_ effect.

**FIGURE 13:**
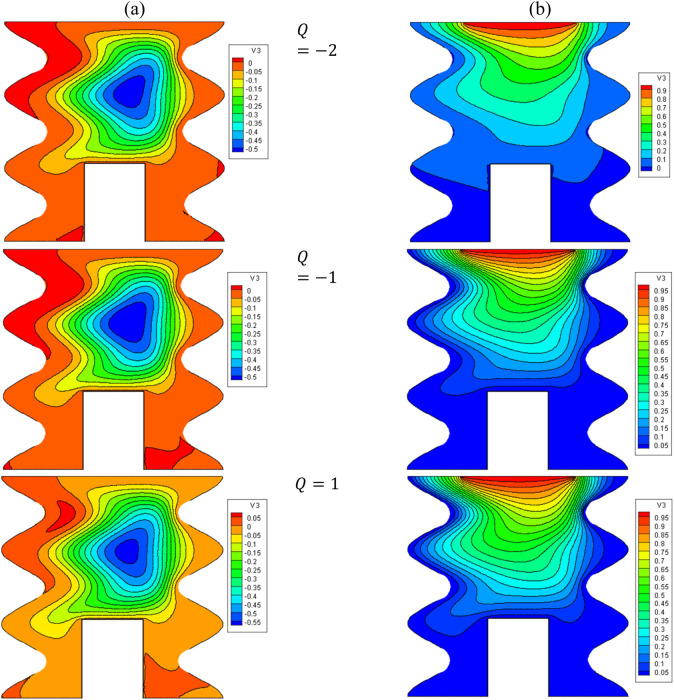
**(A)** Stream function and **(B)** isotherms for HNFs at *ϕ* = 0.05, *Ha* = 10, *B* = 0.5, *D* = 0.5, *R*_*d*_ = 1, *λ* = 3, *α* = 45^0^, *Ra* = 10^5^, Φ = 60^0^, *ϕ*_*Cu*_ = *ϕ*_*Al*_2___*O*_3__ = *ϕ*/2.

**FIGURE 14:**
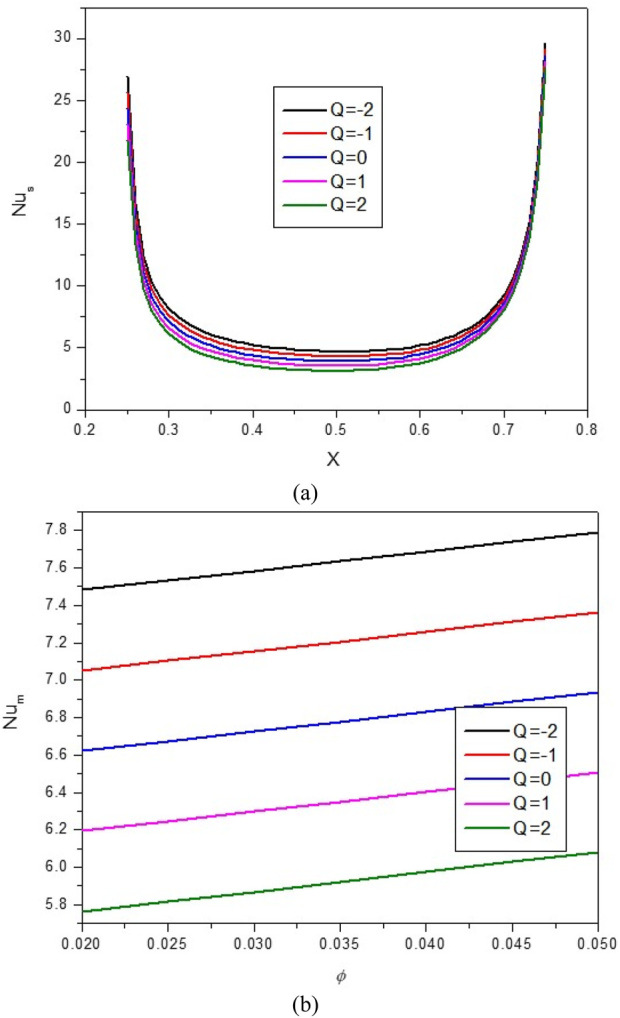
**(A)** Nu and **(B)** Nu_avg_ at *Ha* = 10, *ϕ* = 0.05, *Q* = 1, *B* = 0.5, *R*_*d*_ = 1, *λ* = 3, *α* = 45^0^, *Ra* = 10^5^, Φ = 60^0^, *ϕ*_*Cu*_ = *ϕ*_*Al*_2___*O*_3__ = *ϕ*/2.

Figures 13, 14 show the impact of Q on the stream function, isotherms, and Nu_local_ - Nu_avg_ in specific conditions as follows:

$$\varphi =0.05,B=D=0.5,\text{Ha}=10,R_{d}=1,\lambda =3,\alpha =45{}^{\circ},\text{Ra}=10^{5},\Phi =60{}^{\circ},\varphi_{\text{Cu}}=\varphi_{\text{Al}2O3}=\varphi /2.$$

The results show that when Q = −2, the stream function and isotherms are drawn from the top to the bottom of the cavity, which indicates the predominance of NC and the practical effect of the buoyancy force to circulate the flow. In this condition, the mean temperature of the HNF in the cavity increases, and the Nu_avg_ also increases. When Q > 0, the stream function and isotherms extend towards the side walls, indicating a decrease in the strength of convection and vortices in the cavity and reducing Nu_avg_ and HT. In this regard, by increasing Q from −2 to 2, about a 28% decrease in Nu_avg_ is observed. At a constant Q, by increasing VF from 2% to 5%, about 2.6% increase in Nu_avg_ is observed.

**FIGURE 15:**
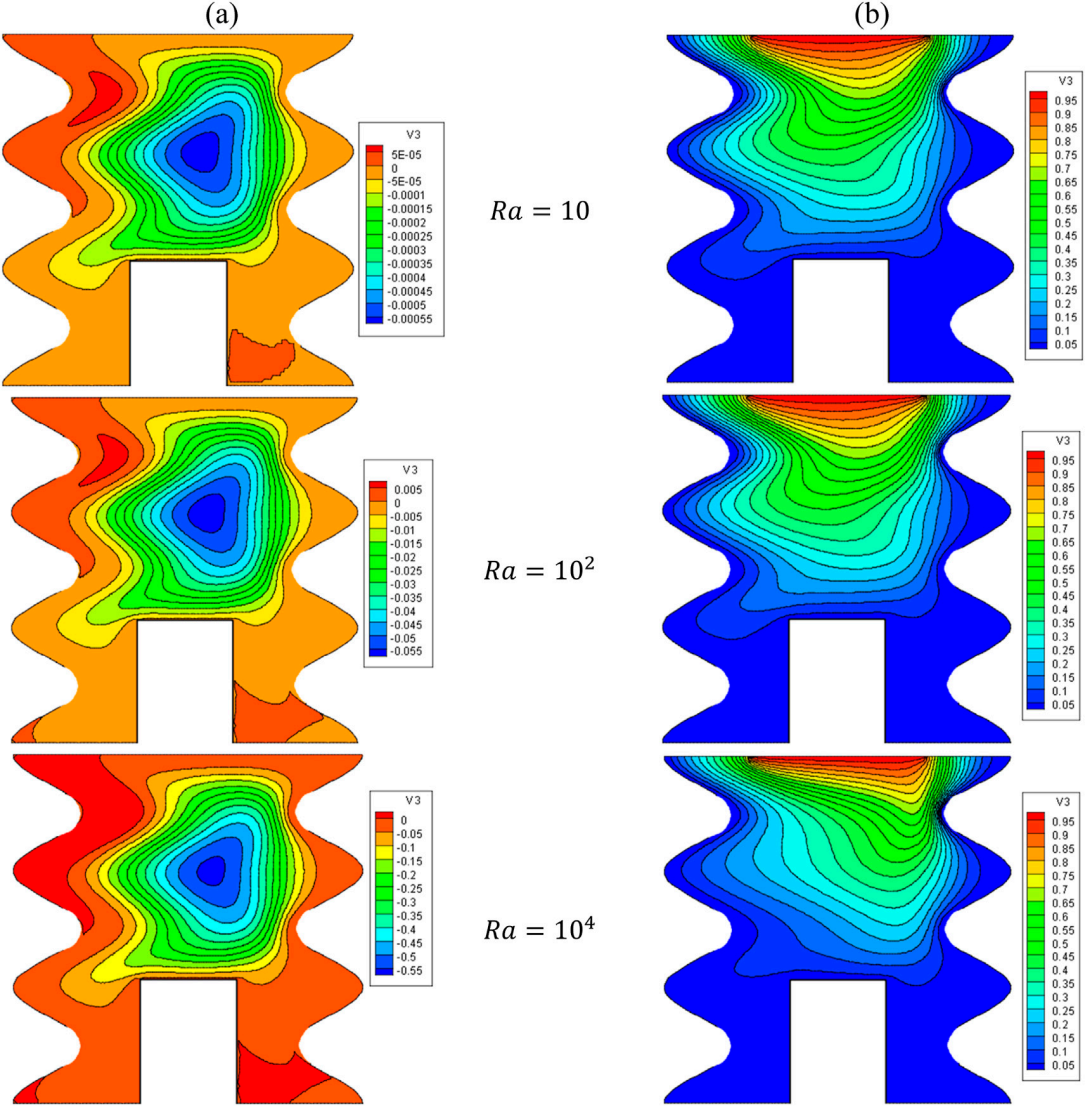
**(A)** Stream function and **(B)** isotherms for HNFs at *ϕ* = 0.05, *Ha* = 10, *B* = 0.5, *D* = 0.5, *R*_*d*_ = 1, *λ* = 3, *α* = 45^0^, *Ra* = 10^5^, Φ = 60^0^, *ϕ*_*Cu*_ = *ϕ*_*Al*_2___*O*_3__ = *ϕ*/2.

**FIGURE 16:**
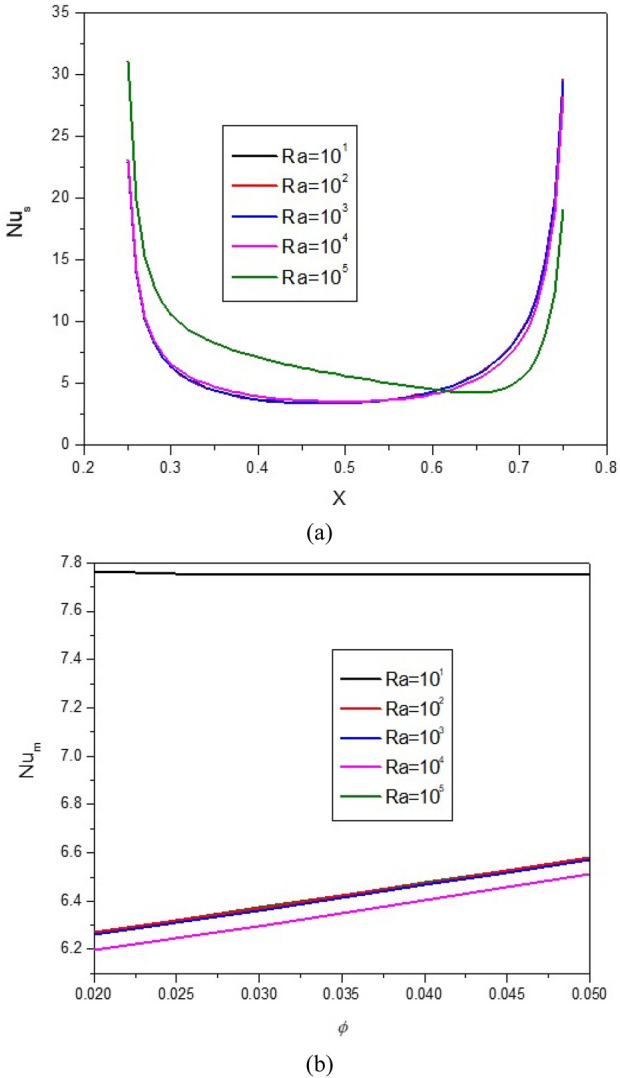
**(A)** Nu and **(B)** Nu_avg_ at *Ha* = 10, *ϕ* = 0.05, *Q* = 1, *B* = 0.5, *R*_*d*_ = 1, *λ* = 3, *α* = 45^0^, *Ra* = 10^5^, Φ = 60^0^, *ϕ*_*Cu*_ = *ϕ*_*Al*_2___*O*_3__ = *ϕ*/2.

Figures 15, 16 illustrate the impact of the Rayleigh number (Ra) on the stream function, isotherms, as well as the local and average Nusselt numbers for $Q=1,B=D=0.5,\text{Ha}=10,R_{d}=1,\lambda =3,\alpha =45{}^{\circ},\Phi =60{}^{\circ},\varphi_{\text{Cu}}=\varphi_{\text{Al}2O3}=\varphi /2$ .

Increasing the Ra from 10 to 10^4^ causes the extension of vortices and swirling flows towards the two cold lateral walls. This issue reduces the mean temperature of the HNF in the cavity and reduces HT. As can be seen, the Nu_avg_ decreases with the increase of the Ra until Ra = 10^4^ and then increases, which can be caused by changing the HT mechanism from conduction to convection. In a constant Ra, by increasing VF from 2% to 5%, about 4.8% increase in Nu_avg_ is observed.

**FIGURE 17:**
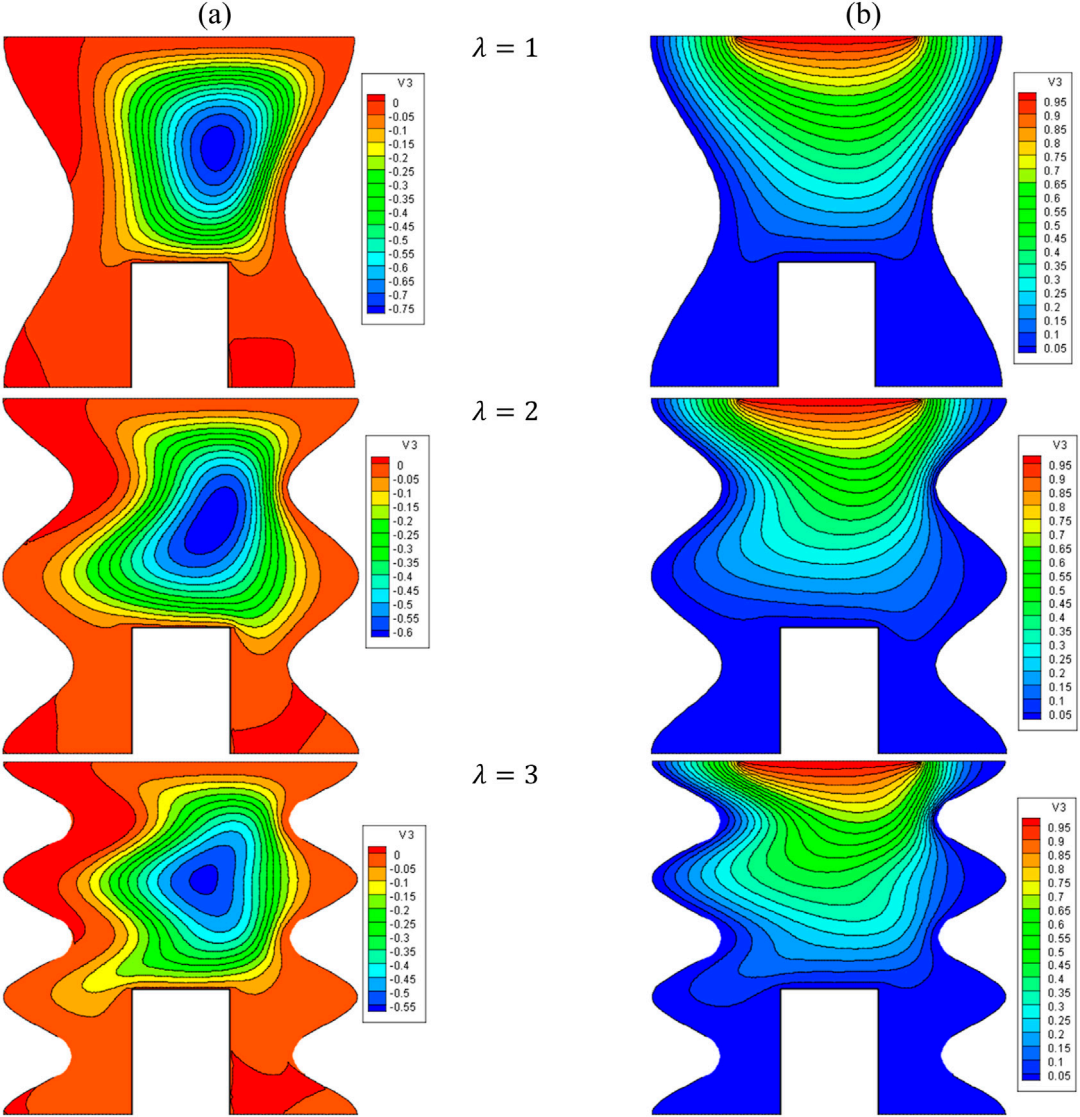
**(A)** Stream function and **(B)** isotherms for HNFs at *ϕ* = 0.05, *Ha* = 10, *B* = 0.5, *D* = 0.5, *R*_*d*_ = 1, *λ* = 3, *α* = 45^0^, *Ra* = 10^5^, Φ = 60^0^, *ϕ*_*Cu*_ = *ϕ*_*Al*_2___*O*_3__ = *ϕ*/2.

**FIGURE 18:**
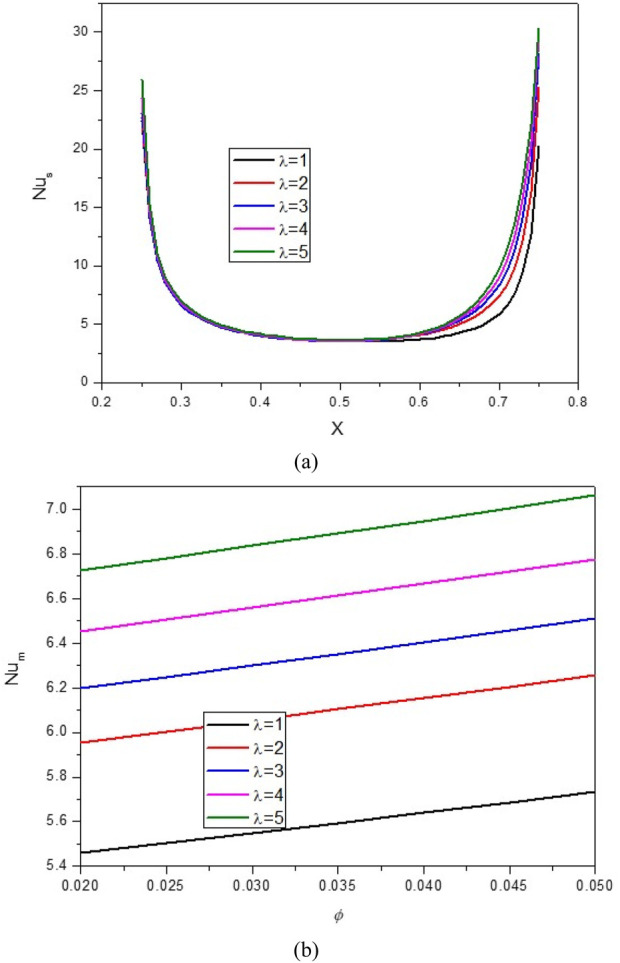
**(A)** Nu and **(B)** Nu_avg_ at *Ha* = 10, *ϕ* = 0.05, *Q* = 1, *B* = 0.5, *R*_*d*_ = 1, *λ* = 3, *α* = 45^0^, *Ra* = 10^5^, Φ = 60^0^, *ϕ*_*Cu*_ = *ϕ*_*Al*_2___*O*_3__ = *ϕ*/2.

Figures 17, 18 demonstrate the impact of wavelength (λ) on the stream function, isotherms, and Nu_local_ and Nu_avg_ for φ = 0.05, Q = 1, B = D = 0.5, Ha = 10, R_d_ = 1, λ = 3, α = 45°, Ra = 10^5^, Ф = 60°, φ_Cu_ = φ_Al2O3_ = φ/2.

It is noticed that by increasing λ, the density of lines increases from the top to the bottom, and the areas of NC become larger. Consequently, the average temperature increases, causing an increase in the Nu_local_ (especially in the lower part of the cavity) and an increase in the Nu_avg_. In this regard, by increasing λ from 1 to 5, about a 28% increase in Nu_avg_ has been observed. It can also be seen that by increasing VF, HT increases so that for each λ, by increasing VF from 0.2% to 0.5%, an increase in Nu_avg_ is observed by about 4.5%.

## 5 Conclusion

In the present numerical research, Al_2_O_3_-Cu-water HNF NC in an inverse U-shaped square wavy PC is studied in the presence of an MF. As NFs are a serious candidate for working flow for electronic devises cooling process this work studied NFs in very novel geometry with radiative porous media. In this research, A, B, D, Ra, Ha, R_d_, Q, and λ are discussed. The remarkable outcomes of the present research are expressed as follows:

• By increasing A from 0.05 to 0.15, about 140%; by increasing D from 0.3 to 0.7, about 94%; by increasing R_d_ from 0 to 5, nearly 775%; and by increasing λ from 1 to 5, about 28%, an increase in Nu_avg_ is observed.
• By increasing B from 0.2 to 0.8, about 20%, by increasing Ha from 0 to 100, about 1.1%, and by increasing Q from −2 to 2, about 28% decrease in Nu_avg_ is observed.
• By increasing Ra from 10 to 10^4^, Nu_avg_ decreases, and then by increasing Ra from 10^4^ to 10^5,^ Nu_avg_ increases.
• By increasing A, the stream function and isotherms compress; by increasing D, the strength of vortices and convection areas increases, and by increasing B and R_d,_ the core size of vortices becomes larger.

As the application of this work is mainly on cooling process of Microelectronic that can save some energy it needs more study in this field.

## Figures and Tables

**Table:** 

**A**	Amplitude
**B**	Dimensionless of heat source/sink length
$B_{0}$	Magnetic field strength
**b**	Length of heat source
$C_{p}$	Specific heat
**C**_**T**_	Difference temperature
**D**	Dimensionless heat source position
Da	Darcy number
d	Location of heat sink and source
g	Acceleration due to gravity
**H**	Length of cavity
**HNF**	Hybrid Nanoofluid
Ha	Hartmann number
K	Permeability of porous medium
k	Thermal conductivity
**k**_**ff**_	Effective thermal conductivity of porous media
**NF**	Nanofluid
${\text{Nu}}_{s}$	Local Nusselt number
${\text{Nu}}_{m}$	Average Nusselt number of heat source
**NC**	natural convection
**MF**	magnetic field
p	Fluid pressure
P	Dimensionless pressure
Pr	Prandtl number
**PC**	porous Cavity
**Q**	heat generation/absorption coefficient
$Q_{0}$	heat generation coefficient
Ra	Rayleigh number
**Rd**	thermal radiation parameter
**S**	Entropy generation
T	Temperature
$T_{c}$	Cold wall temperature
$T_{h}$	Heated wall temperature
u **,v**	Velocity components in *x*, *y* directions
U,V	Dimensionless velocity components
x,y	Cartesian coordinates
X,Y	Dimensionless coordinates
**Greek symbols**
α	Thermal diffusivity
β	Thermal expansion coefficient, $K^{-1}$
ϕ	Solid volume fraction
σ	Effective electrical conductivity
θ	Dimensionless temperature
μ	Dynamic viscosity
ν	Kinematic viscosity
ρ	Density
**ε**	Porosity of the porous medium
**λ**	wavelength
**Subscripts**
c	Cold
0	Reference
f	Pure fluid
h	Hot
m	Average
nf	Nanofluid
**p**	Nanoparticle

## Data Availability

The raw data supporting the conclusions of this article will be made available by the authors, without undue reservation.
